# Strategies Used by Puerto Rican Children in the Cognitive Assessment System and Their Relationship with Planning Performance

**DOI:** 10.3390/jintelligence12090090

**Published:** 2024-09-21

**Authors:** Giselle Cordero-Arroyo, José A. Ramos-Carrasquillo, Imalay M. Cruz-Figueroa, Loggina Báez-Ávila, Manuel Gonzalez-Gonzalez, Mary A. Moreno-Torres, Mario E. Bermonti-Pérez

**Affiliations:** 1 School of Behavioral and Brain Sciences, Ponce Health Sciences University, Ponce 00716, Puerto Rico; joseramos20@stu.psm.edu (J.A.R.-C.); marymoreno@psm.edu (M.A.M.-T.); mbermonti@psm.edu (M.E.B.-P.); 2 Independent Researcher, San Juan 00926, Puerto Rico; imalaycruz21@gmail.com (I.M.C.-F.); loggina.baez@gmail.com (L.B.-Á.); manuelgonzalez3h@gmail.com (M.G.-G.)

**Keywords:** cognitive planning, planning strategies, cognitive assessment system, PASS theory

## Abstract

Studies involving the Cognitive Assessment System (CAS) planning scale typically use only the subtest and scale scores without assessing the strategies employed by the participants. This study addressed this gap and examined the planning strategies used by children in the CAS2: Spanish version and their relationship with planning performance. We conducted an exploratory cross-sectional study with 26 Puerto Rican children aged 8 to 11. Results showed that no strategies were consistently used by participants according to examinees’ reports (*f* = 0–46%), but examiners observed consistent use of some strategies such as “coded left to right, top to bottom”, *f* = 92%; “scanned the page for the next number or letter”, *f* = 100%. Welch’s *t*-tests did not show relationships between participants’ performance and the strategies observed by examiners, | mean differences | = 0.05–0.81, *p*s ≥ 0.05, nor with the strategies reported by participants, | mean differences | = 0.05–1.69, *p*s ≥ 0.05. These findings suggest that although the examiners may observe the use of strategies, the examinees are unaware of the strategies they use, and the strategies used are not associated with their performance. Future studies are needed to confirm these findings.

## 1. Introduction

Several investigations have raised concerns regarding the construct validity of the Cognitive Assessment System (CAS; Keith et al. 2001; Kranzler and Keith 1999; Kranzler et al. 2000). Based on the findings of these studies, researchers concluded that the Planning, Attention, Simultaneous, and Successive (PASS) model does not adequately capture the cognitive constructs measured by the CAS and that these constructs may be more accurately aligned with the Cattell-Horn-Carroll theory (Keith et al. 2001; Kranzler et al. 2000). Among the key findings of these studies are the overlap and relationship between the planning and attention constructs. Additionally, the researchers emphasize that the planning and attention scales primarily measure processing speed or perceptual speed (Kranzler and Keith 1999). Some researchers question whether planning abilities supersede the processing speed component in the planning subtests, particularly when cognitive processes are measured using time measures (Papadopoulos et al. 2018). However, Schneider (2014) argued that the planning scale indeed measures planning within the context of processing speed. According to Schneider, the planning subtests do not prescribe a specific method, allowing individuals to employ their preferred strategies, which can enhance the likelihood of achieving higher scores through efficient strategy utilization. Schneider (2014) recommends that researchers carefully investigate the strategies employed by children during the CAS planning subtests, as such evaluations could help clarify the validity of the planning scale.

However, we have not been able to identify studies with the CAS that examine the strategies children use while taking the CAS planning subtests. Studies involving the CAS are commonly focused on assessment or intervention and typically only use the subtest and scale scores without assessing the strategies employed by the participants. This study addresses this gap by examining the observed and reported cognitive planning strategies used by Puerto Rican children in the Cognitive Assessment System 2: Spanish (CAS2: SP).

### 1.1. Cognitive Planning

According to the Theory of Functional Units proposed by Alexander Luria (1973), planning is the third functional unit of the brain responsible for organizing all conscious activity. The PASS model, which builds on Luria’s theory, defines planning as a neurocognitive ability that enables individuals to determine, apply, self-monitor, and self-correct thoughts and actions to efficiently solve problems or manage situations in both the classroom and everyday life (Naglieri et al. 2014). Planning plays a crucial role in academic success. Researchers suggest that planning mediates students’ motivation, effort, and overall academic performance (Shi and Qu 2022). Effective planning strategies are linked to higher motivation, improved self-regulation, and a greater tendency to engage in productive learning activities (Shi and Qu 2022). Although in their original work Das et al. (1994) mention that the relationship between planning and academic achievement in healthy children is lower compared to successive and simultaneous processing, subsequently they did find that planning is moderately related to academic achievement (i.e., *r* = 0.48; Naglieri et al. 2014). Georgiou et al. (2020) conducted a meta-analysis, finding significant correlations between the CAS planning scale and reading skills (*r* = 0.315 to 0.347) and math skills (*r* = 0.409 to 0.470). However, the authors emphasize that the relationship between PASS processes and academic achievement may be influenced by the language used in the study as well as the specific type of mathematical outcome measured. 

It has been documented that one of the best predictors of academic success is the ability to reflect on our own knowledge (Scorțan 2023). This metacognition is an essential planning component that is characterized by the awareness and understanding of one’s own cognitive processes (Das et al. 1994) and has a crucial role in the use and self-monitoring of strategies.

### 1.2. Planning Strategies in the CAS2

As an important component of planning and metacognitive ability, the CAS2 planning subtest evaluates strategies to solve novel tasks of relatively low difficulty (Naglieri et al. 2014). As measured by the CAS2, planning assists examinees in using effective strategies to complete tasks efficiently and within a specific time frame. Consequently, examinees who employ strategies on the CAS2 planning subtests are more likely to achieve higher scores compared to those who do not (Naglieri and Otero 2024). The CAS2 planning scale subtests include a Strategy Assessment Checklist where the examiner records whether strategies were observed or reported by the examinee during administration. During the tasks, the examiner notes if any strategies from the checklist are used by the examinee and records these in the “observed” column. After completing the three planning subtests, the examinee is asked to explain how they completed each task or page, and these strategies are recorded in the “reported” column. The checklist also includes space to document strategies that are observed or verbally reported but not pre-established. This evaluation of strategies represents a qualitative assessment in the CAS2 that is complementary to the evaluation of the planning ability through the scales and standard scores. 

Although strategy assessment is recognized and incorporated into the planning subtests, Das et al. (1994) mention that its usefulness in reflecting the type of plan or action to be implemented depends on the type of task. They especially highlight the differences between observed strategies and strategies reported by the examinee, noting that the latter are not always informative. Strategies reported by the examinee require greater introspection about one’s cognitive processes. Additionally, some tasks may involve solving problems using automated or well-learned behaviors due to exposure to similar activities in daily life. However, Das et al. (1994) acknowledge that verbally reported strategies can supplement observed strategies during some planning tasks (e.g., Planned Number Matching,) where verbal reports may be most informative for identifying specific strategies. 

### 1.3. Strategy Use in the CAS

In recent years, there has been an increase in articles using the CAS and its various versions and translations worldwide (e.g., Italian: Batini et al. 2024; Chinese: Georgiou et al. 2017; Greek: Georgiou et al. 2024; Spanish: Iglesias-Sarmiento and Pérez 2017; Naglieri et al. 2017). Also, a special volume was published in the Puerto Rican Journal of Psychology titled: “In Search of Justice and Equity in Cognitive Evaluation: Applications of the PASS Theory and the Cognitive Assessment System” (Rodríguez-Arocho and Moreno-Torres 2018). Many of these articles focus on evaluation processes and examine the effectiveness and efficacy of interventions with clinical populations, such as individuals with Attention Deficit/Hyperactivity Disorder and learning disabilities. In reviewing the articles in this special volume, no studies examined the planning strategies. This is consistent with the reviews we have made of the literature in other countries, where we did not find studies that consider the strategies established in the CAS2 Strategy Assessment Checklist. Even in the study conducted by Naglieri and Johnson (2000), in which they examined the effectiveness of a cognitive strategy intervention to improve math calculation based on the PASS theory, they only presented the scores obtained by the participants in the planning scale but did not include nor compare the use of strategies included in the Strategy Assessment Checklist before and after the intervention. 

Studies on the CAS planning scale have primarily evaluated its validity and relationship with processing speed (Haddad 2004; Kranzler et al. 2000; Kranzler and Keith 1999; Papadopoulos et al. 2018). Contributing to the discussion about the validity of the planning scale and its relationship with processing speed, we conducted a study examining the relationship between planning and processing speed (Cordero-Arroyo et al. 2022). Consistent with the findings of the other studies, we found a moderately strong relationship between these two processes (*r* = 0.44). However, similar to previous studies, we focused on subtest and scale scores without evaluating the strategies used during the tasks. Addressing this controversy about the validity of the planning scale, Schneider (2014) analyzed simulated data, generated using the R programming language and incorporated the strategy used. He found a high correlation between the Planned codes subtest scores of the CAS and processing speed (*r* = 0.71). When analyzing the data by grouping observations based on strategy use, the correlations between Planned codes subtest scores and processing speed remained consistent, regardless of whether strategies were accounted for or not (i.e., *r* = 0.74, *r* = 0.71, respectively), suggesting that using strategies does not impact the relationship between processing speed and planning. However, he observed that the average planning score was higher when individuals used more efficient strategies ($\bar{x}$ = 13.5) compared to less efficient strategies ($\bar{x}$ = 8.9). The author stated that if examinees discover and use an efficient strategy, their scores on the planning scale will likely be higher. It is important to mention that Schneider stated that using an “efficient strategy” is equal to “planning”, so we could interpret that using a more efficient strategy suggests better planning ability.

However, speed played a crucial role when the efficiency construct was operationalized in the CAS2 planning subtests. Das et al. (1994) stated that the time required for an examinee to complete a task serves as an indicator of efficiency; this is why CAS2 planning subtests are completed within a specific timeframe. Thus, this could lead to differences in performance on the CAS planning subtests even if the examinees employ strategies deemed equally efficient because of variations in processing speeds. This observation aligns with Schneider’s finding that the relationship between planning and processing speed appears independent of the strategies used. If this is the case, it is worth considering whether planning in the CAS2 is defined and measured as the efficient use of strategies to complete a task quickly.

The Interpretative and Technical Manual of the CAS2 (Naglieri et al. 2014) includes a section on the use of strategies in planning subtests and results from the normative sample on the frequency of strategy use, as well as the relationship between strategy use and the standard scores of planning subtests. They found that observed and reported strategies, as recorded in the CAS Examiner Record Form, increased with age. For instance, at five years old, the frequency was approximately 83%, while from ages eight to 18, it stabilized between 95% and 100%. However, they did not find a relationship between the use of observed and reported strategies and the standard scores of the planning subtests. Although the authors mention that the results “suggest that strategy use was associated with modest improvements in planning scores” (Naglieri et al. 2014, p. 100), the reported effect sizes were trivial (i.e., −0.03 to 0.19). Except for the Planned connections subtest, the average scores were slightly higher (i.e., 0.3 to 0.9) for those who used strategies compared to those who did not. However, this slight increase in score does not represent a substantial change in planning performance.

Aside from Schneider’s (2014) data simulation and the information provided in the CAS2 Interpretative and Technical Manual, we have not been able to identify studies that examine the use of strategies and their relationship with planning performance in the CAS. Research on this topic could enhance the understanding of the validity of the planning scale; hence, our study aims to address this gap.

### 1.4. Study Aim

The current study aimed to examine the cognitive planning strategies used by Puerto Rican children in the Cognitive Assessment System 2: Spanish (CAS2: SP; Naglieri et al. 2017). The specific aims were to: (1) examine the strategies used by children to complete the subtests of the CAS2: SP planning scale, and (2) examine the relationship between the strategies used and the performance in the CAS2: SP planning subtests.

## 2. Methods

### 2.1. Design

We conducted an exploratory cross-sectional design to examine the cognitive planning strategies used by Puerto Rican children in the CAS2: SP. The Strategy Assessment Checklist from the planning scale was used to identify the strategies used by participants to complete the planning subtests. These strategies were also used to examine the relationship between the strategies used and the participants’ performance. Study materials, including data and analysis scripts, are available on the Open Science Framework (OSF) at https://osf.io/ymz84/ (accessed on 29 June 2024).

### 2.2. Participants

The sample size consisted of 26 students with a mean age of 9.46 years (SD = 1.07), who were in third through sixth grade and met the following criteria: (a) spoke Spanish as their native language, (b) were currently enrolled in the participating schools, (c) did not have a mental or neurodevelopmental disorder (e.g., Autism Spectrum, Attention Deficit/Hyperactivity Disorder, Learning Disorders), (d) had uncorrected visual or auditory difficulties, (e) severe motor problems, or (f) current use of psychotropic medications that could affect their task performance.

We implemented a convenience sampling technique in this exploratory study. The original sample size was determined to be 96 participants, but the sample size was adjusted to 30 participants before beginning data collection due to the practical limitations imposed by the COVID-19 pandemic. The final sample comprised 26 children whose data were collected during the academic year of 2022–2023. Table 1 presents participants’ sociodemographic information.

Table 2 shows participants’ performance in the planning scale and subtests. The average score on the planning scale shows that our participants performed just “below average” and that the mean score ranged between “very poor” and “average” classifications.

### 2.3. Recruitment

Permission to conduct our study was obtained from the Institutional Review Board of our institution before data collection and followed the guidelines of the Declaration of Helsinki (protocol code 211107911, on 15 March 2022). Participants were recruited from two private schools in the metropolitan area (i.e., north) of Puerto Rico. The study team provided potential participants with detailed information about the research study and eligibility criteria via in-person or phone meetings. At these meetings, caregivers provided consent and completed the sociodemographic questionnaire via a REDcap online survey. Additionally, children participants agreed to participate in the study synchronously via REDcap before the data collection.

### 2.4. Instrument

We used the Cognitive Assessment System 2: Spanish Version (CAS2: SP), an instrument designed to evaluate the neurocognitive abilities of individuals between the ages of 5 and 18 years. For this study, we used the planning scale of the CAS2: SP and its Strategy Assessment Checklist. The CAS2 defines planning as a neurocognitive ability that enables individuals to determine, apply, self-monitor, and self-correct thoughts and actions to efficiently solve problems or manage situations in the classroom or everyday life (Naglieri et al. 2014). As such, higher scores in these tasks suggest that the individual can maintain an awareness of the need for a solution, monitor how effective it is, solve the initial goal, or consider alternative methods or modifications to their current approach (Flanagan and McDonought 2018). Furthermore, beyond solving novel problems, planning also requires attention, simultaneous processing, and successive processing to employ strategies effectively. The planning scale utilizes three subtests to calculate the individual’s ability to plan: (a) Planned codes, requiring the examinee to fill empty boxes using specific codes; (b) Planned connections, requiring the examinee to connect a series of numbers and letters in a specific order; and (c) Planned numbers, requiring the examinee to find and underline the two numbers that are the same in each row (Naglieri et al. 2014). All the planning subtests must be completed within a time limit, and completion time is used to calculate the participant’s score.

In this study, the Strategy Assessment Checklist of each subtest was used to examine the strategies used by the examinee while completing each task, and the scaled scores on the subtests and the standard scores in the planning scale are used as the indicators of performance. The planning scale has been shown to strongly predict general ability as measured by the Weschler Intelligence Scale for Children—IV, *r* = 0.68, and to moderately predict academic achievement measured by the Woodcock-Johnson—III Total Achievement, *r* = 0.48 (Naglieri et al. 2014). The planning scale has also shown excellent internal consistency, *r* = 0.90, test-retest reliability, *r* = 0.89, and interscorer reliability, *r* = 0.99 (Naglieri et al. 2014).

### 2.5. Data Collection Procedures

Data from each participant was collected in a single 45-min session conducted in an unoccupied and private school office or classroom to avoid unnecessary distractions. These sessions were carried out by a trained doctoral student under the supervision of the primary investigator. Each session was scheduled after class hours or during periods designated by the school’s administrative staff. The 45-min session included a 5-min introduction to complete the assent form and establish rapport with the participant. Once the introduction was completed, the administration of all the planning tasks lasted around 35 minutes, with rest periods provided upon request to minimize fatigue. At the end of each planning subtest, participants were asked questions to explore the strategies they used during the administration, referred to as the reported strategies of the checklist. For example, participants were asked, “Tell me how you did these” or “How did you complete the page?”. To improve the quality of the data, participants were audiorecorded when reporting strategies to facilitate analysis and coding, and the scoring of measures and data entry procedures were double-checked. The final minutes of the sessions were used to answer any questions the participants had.

### 2.6. Statistical Analysis

We analyzed the data using the R environment (R Core Team 2022). Particularly, the *tidyverse* package (Wickham et al. 2019) was used for data wrangling and *gtsummary* to prepare results tables. The number of times participants were observed using or reported using each strategy was used to examine the frequency with which children used each strategy. For this aim, we calculated absolute and relative frequencies and 95% Confidence Intervals (CIs) for the latter using the *gtsummary* package (Sjoberg et al. 2021). We employed the Welch’s *t*-test with bootstrap to examine the relationship between using specific strategies and performance because Quantile-Quantile visualizations revealed asymmetrical data distributions and boxplots revealed outliers. The bootstrap procedure was implemented using the *MKinfer* package (Kohl 2019) with 9999 replications and did not assume a symmetric distribution. We report the means, standard deviations, unstandardized and standardized effect sizes with their 95% CIs, standard errors, and *p*-values adjusted for False discovery rate using the Benjamini/Hochberg procedure (Benjamini and Yekutieli 2001) and assumed two-sided tests and an alpha of 0.05. It is important to note that some strategies were not analyzed because the groups of observations created by grouping data on whether each strategy was used or not were extremely unbalanced with ratios greater than 3:1.

The original sample size of 96 participants was determined based on a sensitivity power analysis that would have made our study reliably detect with 80% statistical power medium effect sizes, *g* = 0.58, assuming *α* = 0.05. The study team deemed this effect size appropriate based on theoretical and practical considerations of the impact that a strategy should have on performance to be considered effective. However, the sample size achieved was 26 participants, which powers our study at 29% to detect the originally estimated effect size.

## 3. Results

Table 3, Table 4 and Table 5 show the results on the frequency with which children used each strategy. We refer to strategies used by more than 50% of the participants as consistently used when describing the prevalence of strategy used. Although cut-off points are always arbitrary, 50% is useful as it references most of the sample. All strategies except for *Said codes to self out loud*, *f* = 7.7%, 95% CI [1.3%, 27%], were consistently observed by examiners during Planned codes, *f* = 54–92%. However, participants did not consistently report any strategy, since the most commonly reported was *Looked for the pattern in the item*, *f* = 38%, 95% CI [21%, 59%]. Examiners observed participants use several strategies consistently during Planned connections, such as *Scanned the page for next number or letter*, *f* = 100%, 95% CI [84%, 100%]; *Lifted the hand off the page to see better*, *f* = 100%, 95% CI [84%, 100%]; and *Looked back at the last letter or number*, *f* = 88%, 85% CI [69%, 97%]. However, participants did not consistently report using any strategy, since the most commonly reported strategy was *Scanned the page for next number or letter*, reported by only *f* = 46%, 95% CI [27%, 66%]. The strategy *Scanned row (either direction) for match*, *f* = 96%, 95% CI [78%, 100%] was consistently observed by examiners during Planned Number Matching, but no strategy was consistently reported by participants. However, *Matched first, then second, number; continued row until match found*; and *Looked at the first, then the last, then the middle number* were relatively common, *f* = 31%, 95% CI [15%, 52%] and *f* = 31%, 95% CI [15%, 52%], respectively.

Table 6 and Table 7 show the results on the relationship between the use of specific strategies and performance on the CAS2: SP planning subtests. We will focus on the magnitude of the differences and de-emphasize statistical significance due to our modest sample size. We include mean differences (MD) and standardized effect sizes *g* to estimate the magnitude of the differences. Our results show only very small differences in performance that were not statistically significant based on the strategies observed by examiners. | MD | < 1, *g* < 0.50, *p*s > 0.05. The largest differences in performance were found for the strategy. *Used a pattern found in a previous item* when observed by an examiner, but this difference was small. MD = −0.82, 95% CI [−2.78, 0.97], *p* = 0.83. The standardized effect sizes showed the largest differences in performance to be for the strategies *Put finger on number and tried to find its match* and *verbalized the numbers*, *g* = −0.38, but this difference was also small. The differences in performance associated with the reported strategies follow a similar pattern showing mostly small and not statistically significant differences in performances based on strategies used, | MD | = 0.05–1.69, *p*s > 0.05. However, the reported strategies show slightly larger differences in performance for strategies. *Repeated the alphabet/number series to self*, | MD | = 1.69, *g* = 0.59, and *scanned the page for the next number or letter*, | MD | = −1.47, *g* = −0.52, but the former implies worst performance when using the strategy.

## 4. Discussion

Studies involving the CAS are commonly focused on assessment or intervention and typically use only the subtest and scale scores without assessing the strategies employed by the participants. This study addressed this gap by examining the cognitive planning strategies (both observed and reported) used by Puerto Rican children in the CAS2: SP. Our findings suggest that Planned codes were the subtest with the most strategies consistently observed, and Planning Number Matching was the one with the fewest strategies consistently observed. These findings could be because at least seven of the 10 descriptions of strategies included in the Strategy Assessment Checklist of the Planning Number Matching subtest are difficult to observe. For example, it is not possible to observe the order in which the examinee looked at the numbers in each string and row (e.g., *looked at first*, *then last*, *then middle number*). Strategies such as this can be recorded and be informative if the examiners report them verbally. We also found very few strategies verbally reported by the participants in the Planned Number Matching subtest. This finding differs from what we expected, considering that Das et al. (1994) mentioned that in the Planned Number Matching subtest, compared with other tasks, the examinee could provide more information about the action plan implemented, due to the number of digits in each string (three to six) and the multiple comparisons that must be made to identify the two numbers that are the same in each row.

One interesting finding was that more strategies can be observed by examiners than reported by participants in the different planning subtests. This finding might be explained by the metacognitive ability of our sample. Metacognition allows us to think about our own mental and learning processes and is strengthened with age (dos Santos Kawata et al. 2021). Das et al. (1994) mention that the metacognitive capacity for planning usually appears at the age of five and is then strengthened until 12 years old, when children begin to use more abstract and analytical thinking. The participants of our study were between eight and 11 years old and might have possessed only a basic level of metacognitive ability that does not allow them to be aware of the relationship between the task and the strategies necessary to perform it efficiently. Therefore, participants could use the strategies in the subtests’ Strategy Assessment Checklist but could not verbally report them. Our results suggest that the strategies observed in the CAS2 could be more useful for assessing children under 12 years of age than the verbally reported strategies.

Another important consideration comes from Simons et al. (2020), who suggested that due to the limited metacognitive capacity that children possess at an early age, they could benefit from explicit instructions for their learning processes and strategies. Based on this suggestion, the questions proposed in the CAS2 could be modified to provide more context and promote metacognitive ability for the reported strategies to be more informative. For example, the instructions *Tell me how you did these* or *How did you complete the page?* could be reformulated to *Tell me how did you found the two pairs of numbers that were the same in each row* to make it more explicit that the intention is for the child to reflect on their action plan. More explicit questions may help children think about their learning process and, as a result, may be of greater benefit to psychologists interested in using these strategies to develop cognitive and educational interventions to strengthen planning ability.

Our results suggest only weak relationships between the strategies used and participants’ performance. These relationships were slightly stronger for strategies reported by examinees, but in some cases, participants performed better when the strategy was not reported. Our results do not align with what Schneider (2014) found while simulating data, where Schneider observed higher scores when efficient strategies were used. On the other hand, our findings are consistent with the results presented by Naglieri et al. (2014) using the standardization sample of the CAS2, where they found trivial effect sizes between the strategies used and planning performance in the three subtests.

It is important to mention that the performance of our sample was lower than expected for healthy participants since their average score on the planning scale was below average. However, when reviewing the literature on studies carried out with the CAS2 on the Puerto Rican population, we identified that the scores in planning range between 82.8 and 102.6 in healthy participants (Báez Ávila 2018; Báez 2019; Cordero-Arroyo et al. 2022; Díaz-Flores et al. 2018; Ortiz-Ortiz 2019; Moreno-Torres et al. 2018). These low scores in healthy children could suggest that the normative data of the CAS2 planning scale are not appropriate for our population, which would require more psychometric studies with this test in Puerto Rico. Another possible explanation could be linked to how planning is taught and promoted in Puerto Rico and how it is implemented, which would require a more extensive and in-depth discussion of cultural differences in planning.

### Limitations and Future Research

We include some limitations of our study that should be addressed in future research. First, our study was an exploratory study with a small sample size. Confirmatory studies with larger samples are needed to validate our findings. Second, only private schools in the metropolitan area (i.e., north) of Puerto Rico were recruited. To address this limitation, future research must diversify the sociodemographic characteristics of the sample (e.g., educational system, region). Third, an interesting pattern that emerged from the data was that strategies were consistently observed more by examiners than reported by examinees. Future studies should explore this pattern, which is consistent with the observation made by Das et al. (1994), but that could not be formally tested in the present study because we had incomplete *information* for the observed-reported pairs of strategies. Fourth, some strategies could not be included in the analyses on the relationship between strategy use and performance because the groups formed by grouping the participants who used versus those who did not use these strategies were extremely unbalanced. A larger sample size could address this limitation by increasing the diversity of strategies used and leading to more balanced distributions. Finally, the performance of our participants was lower than expected for healthy participants since the average score on the planning scale was below the average. It is important to know if these results are consistent in participants with typical performance in the CAS2.

Additionally, future studies should examine Simons et al.’s (2020) proposal to make the instructions for subtests clearer by phrasing the prompts to gather information on strategy use more explicitly. Future studies should examine the moderating role of key variables related to planning ability, such as sex and general ability, which studies suggest impact a person’s metacognitive ability. Also, future studies should employ experimental designs to directly manipulate which strategies are used, leading to more balanced groups of strategy use, increasing internal validity by improving control over confounding variables, and leading to the direct examination of causal relationships.

## 5. Conclusions

The findings of this study have important implications for assessing planning with the CAS2 in clinical and educational settings. Our findings suggest that although examiners may observe the use of strategies when examinees are completing the planning tasks, examinees are not necessarily aware of the strategies they are using. We recommend carefully evaluating the Strategy Assessment Checklist, as some may be difficult to observe. Additionally, it is important to contextualize questions when asking the examinee to explain how they completed the task. Furthermore, the strategies used were not associated with the examinees’ performance. We encourage psychologists to consider the examinee’s age when evaluating planning strategies, as the use of strategies may be limited by metacognitive capacity. Finally, we encourage considering the individual’s processing speed, as it can impact the efficiency in using strategies and, consequently, their planning performance as measured by the CAS2. Research must continue to be carried out to explore the relationship between strategies and planning performance with the CAS2 to provide more evidence and generate new hypotheses. The findings of our exploratory study provide preliminary evidence for continuing the study of planning strategies with CAS2.

## Figures and Tables

**Table 1:** Sociodemographic characteristics of participants.

Variable	N	%
Age		
8	7	26.9
9	4	15.4
10	11	42.3
11	4	15.4
Sex		
Male	10	38.5
Female	16	61.5
School Grade		
Third Grade	7	26.9
Fourth Grade	7	26.9
Fifth Grade	11	42.3
Sixth Grade	1	3.8
Guardian Academic Degree		
High School or less	3	11.5
Technical Degree	9	34.6
Bachelor’s degree	10	38.5
Master’s degree	3	11.5
Doctorate	1	3.8
Household Annual Income		
Less than $50,000	17	65.4
$50,000 to 100,000	8	30.8
100,000 to 20,000	1	3.8
Academic Difficulties		
No	21	80.8
Yes	5	19.2
Behavioral Difficulties		
No	24	92.3
Yes	2	7.7

**Table 2:** Mean, standard deviations, and range on the CAS2: SP planning scales and subtests.

Scale and Subtest	*M*	*SD*	Range
Planning	89.04	9.76	69–106
Planned codes	8.88	2.40	5–15
Planned connections	6.88	2.95	2–13
Planned Number Matching	9.31	1.66	6–12

Note: *N* = 26.

**Table 3:** Observed and reported strategies for Planned codes.

	Observed Strategies		Reported Strategies	
	*N* = 26 ^1^	95% CI ^2^	*N* = 26 ^1^	95% CI ^2^
I Looked for the pattern in the item.				
No	7 (27%)	12%, 48%	16 (62%)	41%, 79%
Yes	19 (73%)	52%, 88%	10 (38%)	21%, 59%
Coded neatly and slowly				
No	12 (46%)	27%, 66%	25 (96%)	78%, 100%
Yes	14 (54%)	34%, 73%	1 (3.8%)	0.20%, 22%
Coded left to right, top to bottom				
No	2 (7.7%)	1.3%, 27%	24 (92%)	73%, 99%
Yes	24 (92%)	73%, 99%	2 (7.7%)	1.3%, 27%
Coded one letter at a time (e.g., did As, then Bs)				
No	3 (12%)	3%, 31%	22 (85%)	64%, 95%
Yes	23 (88%)	69%, 97%	4 (15%)	5.0%, 36%
Looked at codes already completed rather than using the key				
No	7 (27%)	12%, 48%	20 (77%)	56%, 90%
Yes	19 (73%)	52%, 88%	6 (23%)	9.8%, 44%
Said codes to self-out loud				
No	24 (92%)	73%, 99%	26 (100%)	84%, 100%
Yes	2 (7.7%)	1.3%, 27%	0 (0%)	-, -
Used a pattern found in a previous item				
No	8 (31%)	15%, 52%	22 (85%)	64%, 95%
Yes	18 (69%)	48%, 85%	4 (15%)	5%, 36%
Other strategy				
No	21 (84%)	63%, 95%	19 (76%)	54%, 90%
Yes	4 (16%)	5.3%, 37%	6 (24%)	10%, 46%

Note: This study used the CAS2: SP Strategy Assessment Checklist. Missing data for other observed strategy = 1, ^1^ n = sample size, ^2^ CI = Confidence Interval.

**Table 4:** Observed and reported strategies for Planned connections.

	Observed Strategies		Reported Strategies	
	*N* = 26 ^1^	95% CI ^2^	*N* = 26 ^1^	95% CI ^2^
Scanned the page for the next number or letter				
No	0 (0%)	-, -	14 (54%)	34%, 73%
Yes	26 (100%)	84%, 100%	12 (46%)	27%, 66%
Lifted the hand off the page to see better				
No	0 (0%)	-, -	25 (96%)	78%, 100%
Yes	26 (100%)	84%, 100%	1 (3.8%)	0.20%, 22%
Looked back at the last letter or number				
No	3 (12%)	3%, 31%	19 (73%)	52%, 88%
Yes	23 (88%)	69%, 97%	7 (27%)	12%, 48%
Remembered last letter or number				
No	20 (77%)	56%, 90%	23 (88%)	69%, 97%
Yes	1 (3.8%)	0.20%, 22%	3 (12%)	3%, 31%
Repeated the alphabet/number series out loud				
No	21 (81%)	60%, 93%	25 (96%)	78%, 100%
Yes	5 (19%)	7.3%, 40%	1 (3.8%)	0.20%, 22%
Repeated the alphabet/number series to self				
No	21 (81%)	60%, 93%	18 (69%)	48%, 85%
Yes	5 (19%)	7.3%, 40%	8 (31%)	15%, 52%
No strategy				
No	25 (96%)	78%, 100%	26 (100%)	84%, 100%
Yes	1 (3.8%)	0.20%, 22%	0 (0%)	-, -
Other strategy				
No	25 (96%)	78%, 100%	19 (73%)	52%, 88%
Yes	1 (3.8%)	0.20%, 22%	7 (27%)	12%, 48%

Note: This study used the CAS2: SP Strategy Assessment Checklist. ^1^ n = sample size, ^2^ CI = Confidence Interval.

**Table 5:** Observed and Reported Strategies for Planned Number Matching.

	Observed Strategies		Reported Strategies	
	*N* = 26 ^1^	95% CI ^2^	*N* = 26 ^1^	95% CI ^2^
Scanned row (either direction) for match				
No	1 (3.8%)	0.20%, 22%	23 (88%)	69%, 97%
Yes	25 (96%)	78%, 100%	3 (12%)	3.0%, 31%
Looked at the first digit of each number				
No	22 (85%)	64%, 95%	20 (77%)	56%, 90%
Yes	4 (15%)	5.0%, 36%	6 (23%)	9.8%, 44%
Looked at the first, then the last, then the middle number				
No	25 (96%)	78%, 100%	18 (69%)	48%, 85%
Yes	1 (3.8%)	0.20%, 22%	8 (31%)	15%, 52%
Looked at the first, then last, digit of each number				
No	23 (88%)	69%, 97%	22 (85%)	64%, 95%
Yes	3 (12%)	3%, 31%	4 (15%)	5.0%, 36%
Looked at the last, then first, number				
No	25 (96%)	78%, 100%	22 (85%)	64%, 95%
Yes	1 (3.8%)	0.20%, 22%	4 (15%)	5.0%, 36%
Looked at the first two digits of each number				
No	25 (100%)	83%, 100%	19 (76%)	54%, 90%
Yes	0 (0%)	-, -	6 (24%)	10%, 46%
Looked at the last digits to find a match				
No	26 (100%)	83%, 100%	24 (92%)	73%, 99%
Yes	0 (0%)	-, -	2 (7.7%)	1.3%, 27%
Matched first, then second, number; continued row until match found				
No	14 (54%)	34%, 73%	18 (69%)	48%, 85%
Yes	12 (46%)	27%, 66%	8 (31%)	15%, 52%
Put finger on number and tried to find its match				
No	25 (58%)	37%, 76%	25 (96%)	78%, 100%
Yes	11 (42%)	24%, 63%	1 (3.8%)	0.20%, 22%
Verbalized the numbers				
No	15 (58%)	37%, 76%	24 (92%)	73%, 99%
Yes	11 (42%)	24%, 63%	2 (7.7%)	1.3%, 27%
No strategy				
No	26 (100%)	83%, 100%	26 (100%)	83%, 100%
Yes	0 (0%)	-, -	0 (0%)	-, -
Other Strategy				
No	24 (92%)	73%, 99%	24 (92%)	73%, 99%
Yes	2 (7.7%)	1.3%, 27%	2 (7.7%)	1.3%, 27%

Note: This study used the CAS2: SP Strategy Assessment Checklist. Missing data for reported strategy (i.e., “looked at the first two digits of each number”) = 1. ^1^ n = sample size, ^2^ CI = Confidence Interval.

**Table 6:** Observed Strategy Welch’s *t*-test.

Strategy	No	Yes		MD 95% CI					*g* 95% CI	
	M (SD)	M (SD)	MD	LL	UL	SE	*p*	*g*	LL	UL
Coded neatly and slowly	8.83 (1.75)	8.79 (3.02)	0.05	−1.80	1.79	0.90	0.97	0.02	−0.73	0.77
Used a pattern found in a previous item	8.25 (2.25)	9.06 (2.58)	−0.82	−2.78	0.97	0.94	0.83	−0.031	−1.12	0.50
Put finger on number and tried to find its match	9.07 (1.87)	9.73 (1.35)	−0.66	−1.85	0.53	0.60	0.83	−0.38	−1.14	0.38
Matched first, then second, number; continued row until match found	9.29 (1.73)	9.42 (1.68)	−0.14	−1.38	1.11	0.64	0.97	−0.07	−0.82	0.67
Verbalized the numbers	9.07 (1.75)	9.73 (1.56)	−0.65	−1.87	0.58	0.62	0.83	−0.38	−1.14	0.38
Repeated the alphabet/number series to self	6.81 (2.56)	7.40 (3.27)	−0.60	−2.84	1.73	1.15	0.96	−0.20	−0.97	0.57

Note: This study used the CAS2: SP Strategy Assessment Checklist. Estimates are based on bootstrap independent sample Welch’s *t*-test. False discovery rate correction for multiple testing. M = mean, SD = Standard Deviation, SE = Standard Error, MD = Mean Difference, CI = Confidence Interval (Lower limit, Upper limit), *p* = Adjusted *p*-value, *g* = Hedges’ g.

**Table 7:** Reported Strategy Welch’s *t*-test.

Strategy	No	Yes		MD 95% CI					*g* 95% CI	
	M (SD)	M (SD)	MD	LL	UL	SE	*p*	*g*	LL	UL
Looked for the pattern in the item	9.25 (2.65)	8.10 (2.08)	1.15	−0.58	2.96	0.89	0.36	0.455	−0.326	1.23
Looked at the first, then the last, then the middle number	9.56 (1.58)	8.88 (1.89)	0.69	−0.76	2.03	0.71	0.52	0.393	−0.425	1.20
Matched first, then second, number; continued row until match found	9.33 (1.64)	9.38 (1.85)	−0.05	−1.40	1.39	0.71	0.97	−0.024	−0.830	0.783
Scanned the page for next number or letter	6.36 (2.37)	7.83 (3.16)	−1.47	−3.58	0.55	1.06	0.36	−0.518	−1.27	0.248
Repeated the alphabet/number series to self	7.56 (2.83)	5.88 (2.53)	1.69	−0.36	3.78	1.05	0.36	0.592	−0.237	1.41

Note: This study used the CAS2: SP Strategy Assessment Checklist. Estimates are based on bootstrap independent sample Welch’s *t*-test. False discovery rate correction for multiple testing. M = mean, SD = Standard Deviation, SE = Standard Error, MD = Mean Difference, CI = Confidence Interval (Lower limit, Upper limit), *p* = Adjusted *p*-value, *g* = Hedges’ g.

## Data Availability

All data and analysis code can be found in the Open Science Framework’s study repository. Data and analysis code will be made available upon manuscript acceptance for publication.
